# How to choose the surgical side when cerebral blood flow and cerebrovascular response are contradictory in bilateral moyamoya disease?: A case report

**DOI:** 10.1097/MD.0000000000031679

**Published:** 2022-11-11

**Authors:** Mingrui Luo, Jin Yu, Can Xin, Lei Wan, Jianjian Zhang

**Affiliations:** a Department of Neurosurgery, Zhongnan Hospital of Wuhan University, Wuhan, Hubei Province, China.

**Keywords:** case report, cerebrovascular response, hyperventilation test, moyamoya disease, surgical indication

## Abstract

Moyamoya disease (MMD) is a rare cerebrovascular disease characterized by progressive occlusion of the internal carotid artery and the secondary formation of collateral vessels. Bypass surgery is an effective treatment for MMD. Comprehensive evaluation of cerebral blood flow (CBF) and cerebrovascular response (CVR) is the common hemodynamic indication to surgery, the changes of which are usually identical.

**The patient’s main concerns and important examinations:**

We reported a rare case of MMD in a 34-year-old pregnant woman with transient ischemic attacks (TIAs) for 1 month, manifesting as frequent weakness in right limbs for several minutes without obvious cause. The diagnostic digital subtraction angiography (DSA) examination revealed Suzuki Grade I in left side and Grade IV in right side under modified Suzuki scoring. No-hyperventilation test single-photon emission computed tomography (no-HVT SPECT) showed more decreased CBF in the right side of the brain, but HVT SPECT demonstrated a more impaired CVR on the left side. Comprehensively, which side should be operated on is confusing when the changes of CVR and CBF are inconsistent.

**The main diagnosis, therapeutics interventions, and outcomes:**

The patient was diagnosed with bilateral MMD and underwent combined bypass surgery on the left side of the brain. The symptoms of admission were completely relieved after surgery and there were no further cerebrovascular events during the follow-up period of 4 months.

**Conclusion:**

CVR is a primary surgical indication of MMD, especially when the impairment of CVR and CBF are not consistent in the ipsilateral hemisphere. Meanwhile, HVT is the vital vasoactive challenges test for measuring CVR in MMD.

## 1. Introduction

Moyamoya disease (MMD) is a chronic cerebrovascular disorder that is characterized by progressive occlusion of distal part of internal carotid artery (ICA) and proximal part of middle and anterior cerebral arteries which are main branches within the circle of Willis, with arterial collateral vessels at the base of the brain.^[1]^ At present, no evidence supports that drug treatment is able to delay or reverse the progression of MMD.^[2]^ Generally accepted indications for surgical revascularization include the following: clinical symptoms for either ischemic or hemorrhagic strokes with decreased cerebral blood flow (CBF), cerebrovascular response (CVR).^[3,4]^ At the same time, the impairment of the cerebral hemodynamic indexes in MMD are always consistent. However, when the impairment of CBF and CVR are inconsistent, for example, CBF decreased in in 1 hemisphere while CVR decreased in another 1, it is difficult to decide which side should be operated on. CBF may not accurately reflect cerebral hemodynamics due to the presence of compensatory vessels in MMD. CVR denotes the ability of cerebral vessels to dilate or constrict in response to challenges or maneuvers.^[5]^ A study of adults with MMD showed that CVR impairment detected preoperatively was an independent risk factor for severe ischemic events in MMD patients after cerebrovascular bypass.^[6]^ Various imaging techniques, such as positron emission tomography, single-photon emission CT (SPECT) and transcranial doppler, have been widely used in combination with vasoactive challenges to map CVR. Hyperventilation test (HVT) means that volunteers instructed to breathe deeply at a respiratory rate of 24 respirations per minute,^[7]^ which is increasing used to measure CVR in vasoactive challenges.

We presented a case that a 34-year-old female with MMD who underwent surgical treatment based on CVR measured by HVT SPECT (which refers to that SPECT combines with HVT) got a good result. This will provide an important reference for future surgical indications.

## 2. Case report

A 34-year-old female patient was admitted to our hospital referred by physician on July 2021 owing to transient ischemic attacks (TIAs) for 1 month, manifesting as frequent weakness in right limbs for several minutes without obvious cause. A physical examination revealed that the patient was conscious and able to answer questions accurately. The patient has not suffered other past medical, surgical history or other histories.

**Figure 1.**
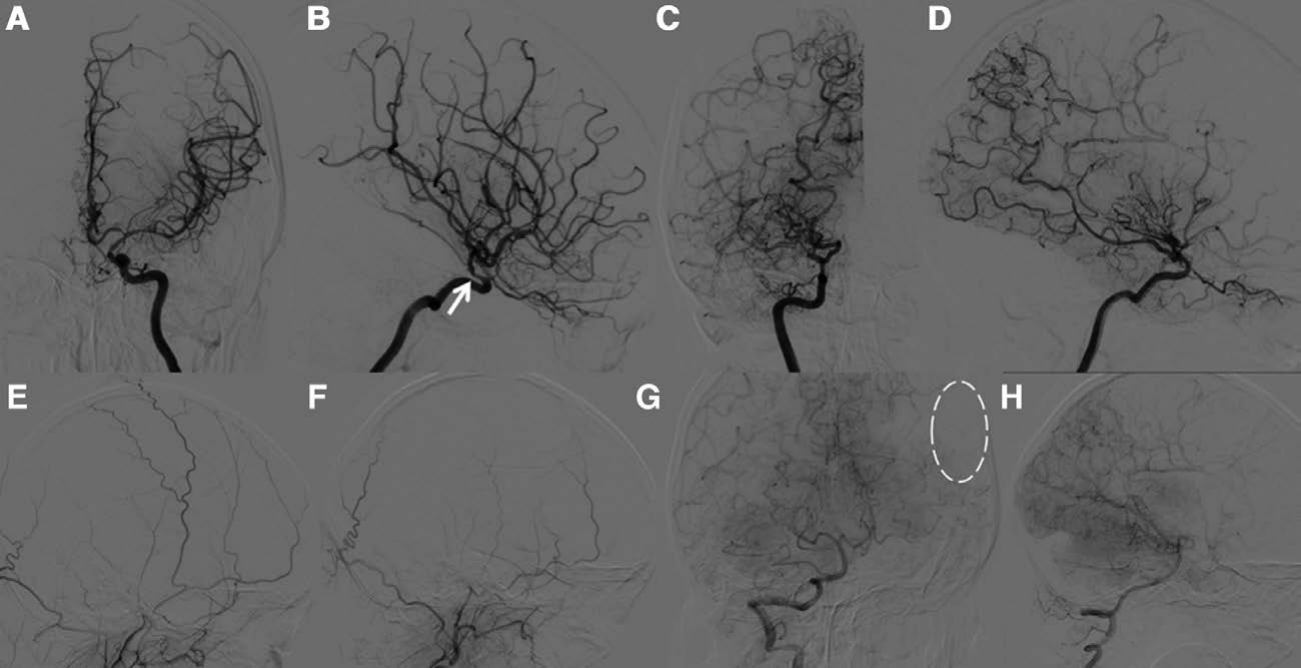
Preoperative diagnosis of MMD. (A–B) Preoperative DSA showed mild stenosis (white arrow) of the terminal portions of left ICA; (C–D) Preoperative DSA showed occlusion of the MCA and the occurrence of abundant moyamoya vessels in the brain base; (E–F) Preoperative DSA revealed respectively non-compensatory vessel formation in left and right ECA; (G–H) Preoperative DSA revealed significant compensatory vessels on the right side, but not on the left (dotted circle) in VA. DSA = digital subtraction angiography, ECA = external carotid artery, ICA = internal carotid artery, MMD = moyamoya disease, MCA = middle cerebral artery, VA = vertebral artery.

**Figure 2.**
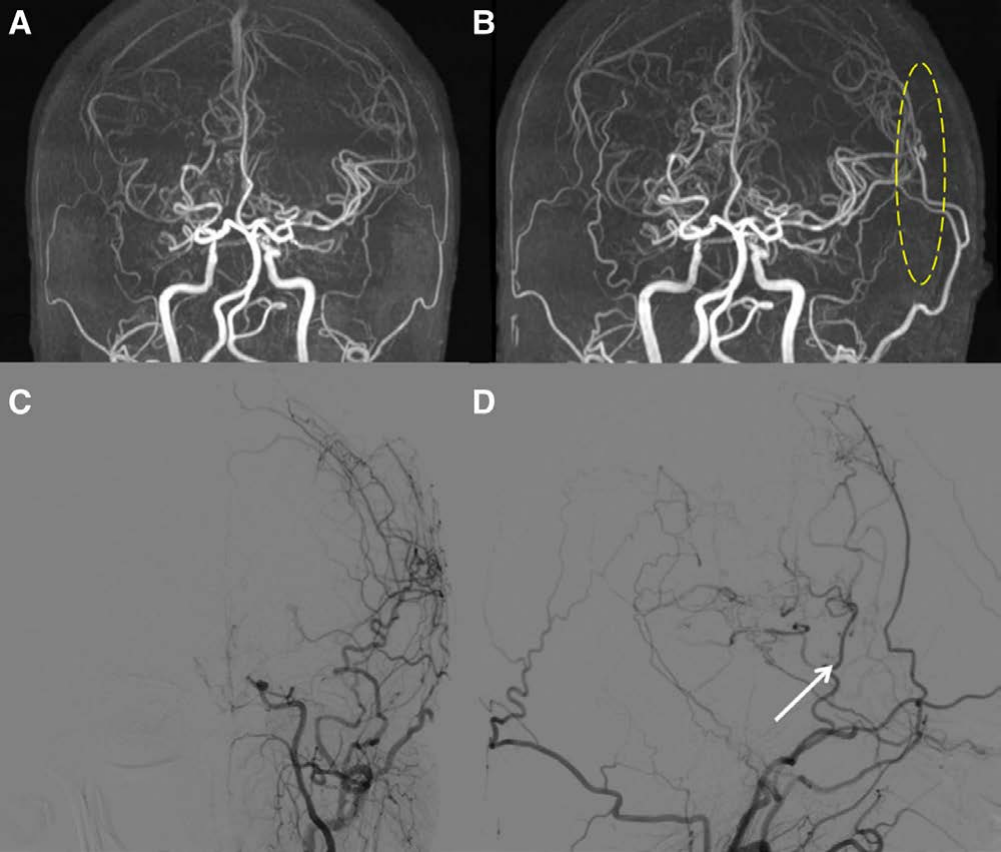
(A) Preoperative MRA showed mild stenosis of the terminal portions of left ICA and occlusion of the MCA; (B) MRA showed the change of left STA-MCA bypass 1 day after surgery (dotted circle); (C–D) DSA showed occlusion of the left ICA in a short period and patency of the left STA-MCA bypass 3 months after surgery (white arrow). DSA = digital subtraction angiography, ICA = internal carotid artery, MRA = magnetic resonance angiography, STA-MCA = superficial temporal artery–middle cerebral artery.

**Figure 3.**
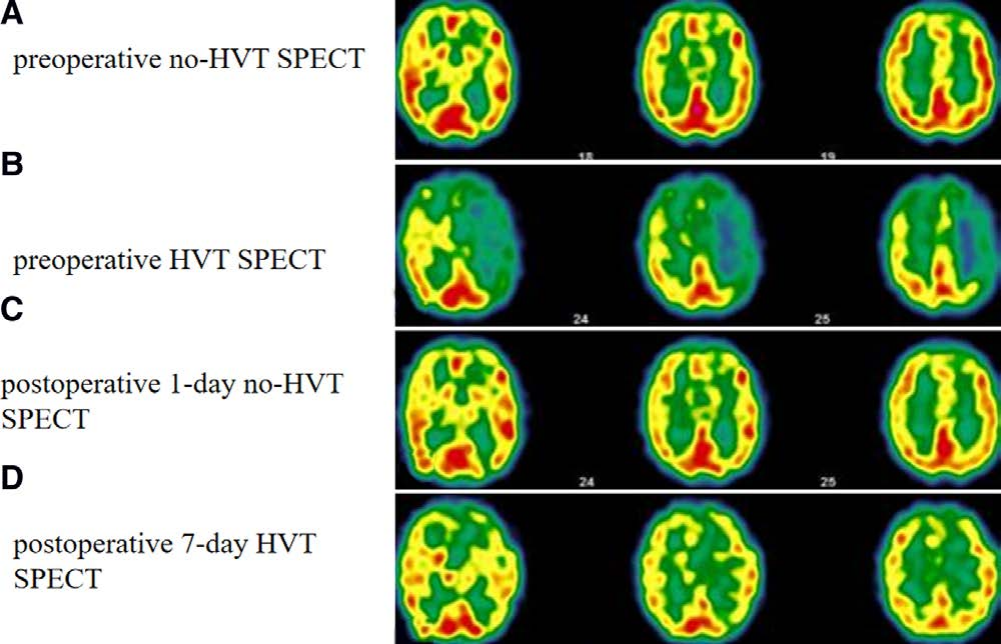
Temporal profile of 123I-IMP-SPECT images showed: (A) decreased CBF in bilateral frontal parietal lobe and right temporal lobe; (B) more decreased CBF in the left frontal, temporal, parietal lobes and basal ganglia than that of the contralateral hemisphere; (C) no obvious changes compared to preoperative no-HVT SPECT; (D) a significant improvement, compared to preoperative no-HVT SPECT. CBF = cerebral blood flow, HIT = hyperventilation test, SPECT = single photon emission computed tomography.

Following admission, a digital subtraction angiography (DSA) examination revealed Suzuki Grade I in left side and Grade IV in right side under modified Suzuki scoring (mSS) (Fig. 1A–D),^[8]^ compensatory collateral formed between intracranial and extracranial vessels (Fig. 1E–H), associated with magnetic resonance angiography (MRA) results (Fig. 2A), suggested bilateral MMD. Preoperative baseline SPECT (which refers to “no-HVT SPECT”) showed more decreased CBF in the right side of the brain, compared to the left side (Fig. 3A). Routine preoperative blood gas analysis (BGA) showed carbon dioxide partial pressure (PaCO_2_) of 38.5 mm Hg. We amazingly found that the patient developed weakness and aphasia for about 15 minutes after 4 minutes when she was breathe deeply at a respiratory rate of 24 respirations per minute, and the following SPECT (which refers to “HVT-SPECT”) immediately demonstrated a more impaired CVR on the left side (Fig. 3B). BGA after HVT showed PaCO_2_ of 27.4 mm Hg.

**Figure 4.**
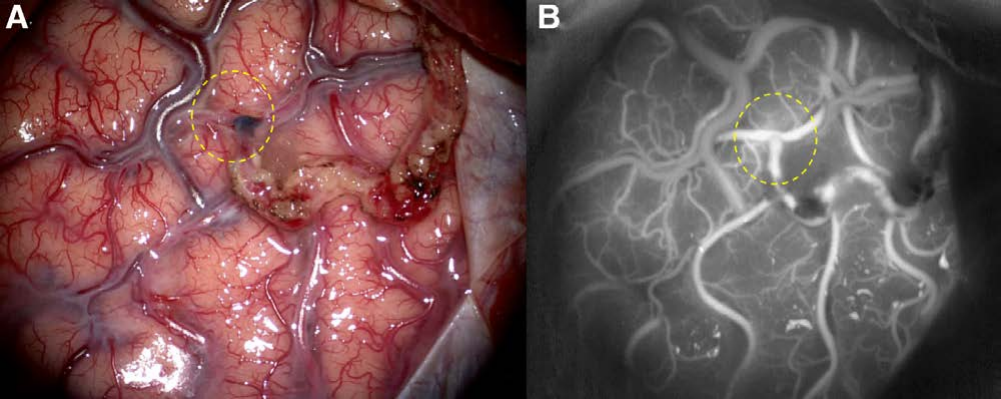
Surgical view of left STA-MCA anastomosis. STA-MCA anastomosis with EDMS in the left hemisphere (the temporal lobe area) was performed. (A) The MCA (M4 segment) was anastomosed to STA; (B) Indocyanine green video-angiography demonstrated apparent patent bypass with the favorable distribution of bypass flow. The yellow circles showed the anastomotic site. EDMS = encephalo-duro-myo-synangiosis, STA-MCA = superficial temporal artery–middle cerebral artery.

According to higher Suzuki stage and more decreased CBF in the right side, it should have been operated on preferentially. Due to more impaired CVR on the left side, superficial temporal artery–middle cerebral artery (STA-MCA) anastomosis (Fig. 4A) with encephalo-duromyo-synangiosis in the left hemisphere (the temporal lobe area) was instead performed by a senior neurosurgeon (JJZ) with more than 10 years bypass experience. The detailed surgical procedures were already described elsewhere.^[9]^ The patency of the bypass was promptly confirmed by indocyanine green video-angiography (Fig. 4B).

Accompanied by the strict administration of fluid intake and blood pressure after surgery, the patient did not display additional neurological deterioration during or after surgery. MRA showed an apparently patent bypass 1 day after surgery (arrow in Fig. 2B). The postoperative 1-day no-HVT SPECT result (Fig. 3C) showed no obvious changes, but the postoperative 7-day HVT SPECT (Fig. 3D) showed a significant improvement, compared to preoperative no-HVT SPECT. The patient was discharged 7 days after surgery. Three months after operation, DSA showed occlusion of the left ICA (Fig. 2C), the patency of left STA-MCA bypass and compensatory collateral formed in extracranial vessels (Fig. 2D).

The symptoms of admission and the symptoms under HVT were completely relieved after surgery, and there were no further cerebrovascular events during the follow-up period of 4 months.

## 3. Discussion

The main symptoms of MMD are hemorrhage and ischemia. Ischemic events, which manifest themselves in a variety of ways, such as stroke, hemiplegia and dizziness, are the most important clinical manifestation of MMD. Whether it manifests itself depends on the location and size of cerebral ischemia focus. However, MMD is a chronic occlusive cerebrovascular disease, which has relative stable cerebral hemodynamics in quiet conditions. The real condition of intracranial hemodynamics can only be revealed under cerebrovascular vasoactive challenges.

Vasoactive challenges include carbon dioxide (CO_2_) inhalation, breath holding test, acetazolamide (ACZ) administration and HVT. The reliability of CO_2_ inhalation tests has been widely recognized, but some patients may experience discomfort or even adverse cardiovascular reactions after inhaling CO_2_. Breath holding test is simple and feasible, but not accurate. ACZ has a complex vasodilation mechanism and the duration of vasodilation is long, which may lead to related complications. Saito et al reported that 63% of patients would experience adverse reactions of various symptoms such as headache, dizziness, numbness of limbs and weakness after 1 to 3 hours of ACZ challenge, which could last up to 72 hours.^[10]^ HVT could induce a drop in PaCO2 and even hypocapnia in the brain through deep and fast breath, contracting cerebral vessels. Generally, HVT should stop when it lasts for more than 5 minutes without special symptoms or symptoms of insufficient cerebral blood supply occur,^[7]^ then SPECT will be performed immediately. In our concept, HVT is more feasible and safer than the above modalities. Because it can be halted by the patients themselves at the onset of ischemic symptoms, then avoiding the occurrence of a cerebral infarction. Thus, it could be the best method to evaluate the CVR in vasoactive challenges tests at this point.

CVR is defined broadly as the ability of brain parenchyma to adjust CBF in response to altered metabolic demand or a vasoactive stimulus. It has been reported that CVR impairment is an independent risk factor for stroke.^[11]^ Kuroda et al found CVR can better reflect the collateral circulation for patients with occlusion of ICA, so as to accurately predict the risk of cerebrovascular diseases caused by insufficient blood supply.^[12]^ Thus, collateral circulation plays an important role in CVR. For TCD measurements of the cerebral circulation, a distinction is made between primary and secondary collateral pathways.^[13]^ Primary collaterals are considered the anterior communicating artery and posterior communicating artery as part of the circle of Willis, whereas secondary collaterals include the ophthalmic artery and the more distal leptomeningeal collaterals.^[14]^ The latter is thought to be only recruited when the primary collaterals cannot be drawn upon or are insufficient to compensate the blood flow demand.^[15]^ Compensatory neovascularization plays an important role in recanalization of occluded vessels as compensation for the tertiary collateral pathway.^[16–19]^ Busc et al found that CVR can be improved and the occurrence of cerebral infarction can be prevented through the formation of intracranial collateral arteries in the mouse model of chronic ischemia.^[20]^ Based on DSA in this case, the patient had abundant vascular compensation of the posterior circulation on the right side of the brain and regenerative moyamoya vessels in the brain base, indicating a less severe degree of CVR impairment on the right side, consistent with the preoperative HVT-SPECT results. In addition, hemispheric steal phenomenon may also explain the left-right difference in CVR. Pistolese et al found that there was a hemispheric steal phenomenon during hyperventilation in cerebrovascular diseases.^[21]^ Negative CVR, also known as steal phenomenon, occurs when a stimulus results in the redistribution of blood flow from regions of exhausted cerebrovascular reserve to areas with preserved vasodilatory capacity.^[22]^ In this case, the patient had a more stable hemodynamic state on the right side of the brain. When the left ICA stenosis was relieved by a stimulus, more blood flow may pass through the anterior communicating artery into the right hemisphere with lower resistance, resulting in hypoperfusion on the left side.

Consequently, the patients will face a higher risk of developing cerebral infarction on the left side on the future. As a result, we performed bypass surgery on the left side rather than on the right side which had an advanced Suzuki stage and the patient received satisfactory results. Furthermore, we surprisedly found that there was a complete occlusion of the left MCA in patient with MMD only 3 months after surgery (Fig. 2C). In my opinion, there are 2 explanations for it. Firstly, the MMD may be in a rapidly progressive stage and the patient may have ischemic events if bypass surgery is not performed as soon as possible. Secondly, the replacement of the ipsilateral intracranial vessels by the bypass vessel. Therefore, Impaired CVR measured by HVT SPECT may be a predictor to rapid progression of MMD.

## 4. Conclusion

CVR is a primary surgical indication of MMD, especially when the impairment of CVR and CBF are not consistent in the ipsilateral hemisphere. Meanwhile, HVT is the vital vasoactive challenges test for measuring CVR in MMD.

## Acknowledgments

The authors thank all participants in the study.

## Author contributions

Conceived the project, designed the project, analyzed data, and drafted the manuscript: Mingrui Luo and Jianjian Zhang. Extract and analyzed data: Jin Yu, Can Xin and Lei Wan. Designed the project, edited the manuscript and approved the final version: Jianjian Zhang.

**Conceptualization:** Mingrui Luo.

**Data curation:** Mingrui Luo.

**Funding acquisition:** Jianjian Zhang.

**Investigation:** Can Xin, Jianjian Zhang.

**Resources:** Jin Yu.

**Software:** Lei Wan.
